# Serotype conversion gene *rfbT* is directly regulated by histone-like nucleoid structuring protein (H-NS) in *V. cholerae* O1

**DOI:** 10.3389/fmicb.2023.1111895

**Published:** 2023-02-01

**Authors:** Yu Han, Jing Li, He Gao, Xiaorui Li, Ran Duan, Qian Cheng, Biao Kan, Weili Liang

**Affiliations:** State Key Laboratory of Infectious Disease Prevention and Control, National Institute for Communicable Disease Control and Prevention, Chinese Center for Disease Control and Prevention, Beijing, China

**Keywords:** *rfbT*, H-NS, *V. cholerae*, transcriptional regulation, serotype shift

## Abstract

*Vibrio cholerae* serogroup O1 (*V. cholerae* O1) is closely associated with cholera epidemics and has two main immunologically distinguishable serotypes, Ogawa and Inaba. Isolates serotype as Ogawa if the O-antigen polysaccharide (O-PS) is methylated or as Inaba if the O-PS is not methylated. This methylation is mediated by a methyltransferase encoded by the *rfbT* gene, and the mutation and low expression of *rfbT* results in serotype switch from Ogawa to Inaba. Previously, we have shown that cAMP receptor protein (CRP) activates *rfbT*. In this study, we demonstrated that histone-like nucleoid structuring protein (H-NS) is directly involved in the transcriptional repression of *rfbT*. This finding is supported by the analyses of *rfbT* mRNA level, *rfbT*-*lux* reporter fusions, electrophoretic mobility shift assay (EMSA), and DNase I footprinting assay. The *rfbT* mRNA abundances were significantly increased by deleting *hns* rather than *fis* which also preferentially associates with AT-rich sequences. A *single-copy chromosomal complement of hns partly restored the down-regulation of rfbT*. Analysis of *rfbT*-*lux* reporter fusions validated the transcriptional repression of *hns.* Subsequent EMSA and DNase I footprinting assay confirmed the direct binding of H-NS to *rfbT* promoter and mapped the exact binding site which was further verified by site-directed mutagenesis and promoter functional analysis. Furthermore, we found that in *hns* deletion mutant, CRP is no longer required for transcriptionally activating *rfbT*, suggesting that CRP functions as a dedicated transcription factor to relieve H-NS repression at *rfbT*. Together, this study expanded our understanding of the genetic regulatory mechanism of serotype conversion by global regulators in *V. cholerae* O1.

## 1. Introduction

Cholera is an acute watery diarrheal disease caused by *Vibrio cholerae*, which is naturally present in the environment and autochthonous to coastal and estuarine ecosystems. People are usually infected by ingesting *V. cholerae*-contaminated water or food. Upon colonization of the host, *V. cholerae* produces cholera toxin (CT), which acts on intestinal epithelial cells, resulting in secretory diarrhea and even death within a few days if without treatment (Chiang and Mekalanos, 2000). Based on the heat-stable somatic O antigen, the species *V. cholerae* is divided into more than 200 serogroups. Among them, only two serogroups, toxigenic O1 and O139, have been demonstrated to cause epidemic and pandemic cholera. O1 serogroups have caused seven pandemics, and O139 emerged in the seventh pandemic (Longini et al., 2002). Serogroup O1 has two biotypes, El Tor and classical. The first six pandemics are believed to be caused by the classical biotype, whereas the seventh pandemic that started in the early 1960s is caused by the El Tor biotype.

*Vibrio cholerae* O1 antigen consists of at least three types of antigenic factors: A, B, and C. According to the differences of the antigenic factors, each of the two biotypes can be further classified into two major cross-reacting serotypes, Ogawa and Inaba. Ogawa serotype strain expresses A and B antigens as well as a small amount of C antigens, while Inaba only expresses A and C antigens. A third serotype, Hikojima expressing both the B and C antigens, is also reported but is rare and unstable (Chatterjee and Chaudhuri, 2003). Ogawa and Inaba serotypes differ only by a single 2-*O*-methyl group that is present in the upstream (nonreducing) terminal perosamine unit of the Ogawa O-antigen polysaccharide (O-PS) but is absent in Inaba (Chatterjee and Chaudhuri, 2003). An isolate is serotyped as Ogawa if its O1 serogroup O-PS is methylated and as Inaba, if its O-PS is not methylated (Ito et al., 1993). This methylation is catalyzed by a methyltransferase encoded by the *rfbT* gene (also known as *webT* or *tsfB*; Rijpkema et al., 2004). Therefore, genetic alterations of *rfbT* resulted from various mutational events, such as specific point mutation, single nucleotide or short fragment insertion/deletion, or transposase insertion can all lead to the serotype shift from Ogawa to Inaba (Sharifnia et al., 2012; Liang et al., 2013; Karlsson et al., 2016). Once the complete *rfbT* is replenished, the Inaba type can be reverted to the Ogawa.

The serotype shifts can occur during subculture *in vitro*, passage *in vivo*, or even during pandemics (Ito et al., 1993; Liang et al., 2013; Karlsson et al., 2016). Recently, a major serotype switch (ranging from 7 to 100%) from Ogawa to Inaba was discovered after 5 years of the onset of cholera in Haiti in October, 2010 (Alam et al., 2016). Such serotransitions are nonrandom processes and thought to be related to selective pressures of serotype-specific immunity within the host population or environmental stress, the specific intrinsic drivers and regulation mechanism remain to be investigated (Longini et al., 2002; Karlsson et al., 2016). Previously, we demonstrated that a global regulator, cAMP receptor protein (CRP), positively regulates *rfbT* transcription through directly binding to a non-canonical CRP binding site (CBS) in its promoter region (Li et al., 2019).

The histone-like nucleoid structuring protein (H-NS) is a global regulator of environmentally controlled gene expression. It belongs to a small family of nucleoid-associated proteins (NAPs; Winardhi et al., 2015). This family comprises a group of basic, low molecular weight DNA binding proteins that participate in chromatin organization, restraining of DNA supercoiling, and transcription regulation. The factor for inversion stimulation (Fis), leucine-responsive protein (Lrp), heat-labile protein (HU) and integration host factor (IHF) are all members of this family. H-NS consists of a coiled-coil N-terminal domain that mediates the protein oligomerization and a C-terminal DNA-binding domain, which binds to promoters exhibiting AT-rich and highly curved regions as transcriptional inhibitors, affecting a broad spectrum of physiological processes including virulence-related genes at multiple phases of the *V. cholerae* life cycle (Winardhi et al., 2015; Ayala et al., 2017). Totally 701 genes have been identified to be regulated by H-NS in *V. cholerae* (Wang et al., 2015).

In this study, we show that H-NS negatively regulates the transcription of the serotype-switching gene *rfbT* by directly binding to its promoter region, whereas *fis* does not affect its expression. CRP likely activates transcription of *rfbT* through derepression of H-NS.

## 2. Materials and methods

### 2.1. Bacterial strains, culture conditions, and plasmids

Bacterial strains and plasmids used in this study are shown in Table 1. The *V. cholerae* O1 El Tor biotype, Ogawa serotype strain C7258 was used as wild-type (WT) precursor (Peru isolate, 1991). The mutant Δ*crp* (WL7258) was generated previously (Li et al., 2019). *E. coli* DH5αλ*pir* and S17-1λ*pir* were, respectively, used for cloning and conjugation purposes, ER2566 is used as host for the expression and purification of *hns* cloned into the pTXB1 vector. All strains were grown in Luria–Bertani (LB) broth (Oxoid, Basingstoke, United Kingdom) containing 1% NaCl (170 mM) at 37°C. When necessary, culture media were supplemented with ampicillin (Amp, 100 μg/ml), chloramphenicol (Cm, 10 μg/ml for *E. coli*, 2.5 μg/ml for *V. cholerae*) or polymyxin B (100 units/ml). Isopropyl-β-D-thiogalactopyranoside (IPTG) was used at a concentration of 0.5 mM for induction purposes.

**Table 1:** Strains and plasmids used in this study.

Strains/plasmids	Characteristics	References/sources
***E. coli***		
S17-1*λpir*	*thr thi tonA leu supE lacY recA*:: RP4-2Tc:: Mu (*λpir*R6K)	Lab stock
DH5α*λpir*	F-D(*lacZYA-argF*)U169 *recA endA1 supE44 relA1λ::pir*	Lab stock
ER2566	*fhuA2 lacZ::T7 gene1* [*lon*] *ompT gal sulA11 R*(*mcr*-7*3*:: *miniTn*10--Tet^S^)2 [*dcm*] *R*(*zgb-210::Tn10*--Tet^S^) *endA1* Δ(*mcrC-mrr*)*114::IS10*	Lab stock
***V. cholerae***		
C7258	Wild-type, El Tor biotype	Peru isolate, 1991
Δ*crp* (WL7258)	C7258, *crp* deletion mutant	Liang et al. (2007)
Δ*hns*	C7258, *hns* deletion mutant	This study
Δ*crp*Δ*hns*	C7258, *crp* and *hns* deletion mutant	This study
C7258Δ*hns*::*hns*	pGRG25 chromosome complementation	This study
Δ*fis*	C7258, *fis* deletion mutant	This study
Δ*crp*Δ*fis*	C7258, *crp* and *fis* deletion mutant	This study
C7258/pBBR*lux-rfbT*1	pBBR*lux*-*rfbT*1 in C7258	This study
Δ*hns* /pBBR*lux-rfbT*1	pBBR*lux*-*rfbT*1 in Δ*hns*	This study
C7258/pBBR*lux-rfbT*2	pBBR*lux*-*rfbT*2 in C7258	This study
Δ*hns*/pBBR*lux-rfbT*2	pBBR*lux*-p*rfbT*2 in Δ*hns*	This study
C7258/pBBR*lux-rfbT*1-M1	pBBR*lux-rfbT*1-M1 in C7258	This study
C7258/pBBR*lux-rfbT*1-M3-1	pBBR*lux-rfbT*1-M3-1 in C7258	This study
C7258/pBBR*lux-rfbT*1-M3-2	pBBR*lux-rfbT*1-M3-2 in C7258	This study
Δ*hns*/pBBR*lux-rfbT*1-M3-2	pBBR*lux-rfbT*1-M3-2 in Δ*hns*	This study
***Plasmids***		
pWM91	Suicide vector containing R6K ori, *sacB*, *lacZα*; Amp^R^	Lab stock
pWM91-*hns*	1.6 kb *BamH*I-*Spe*I Δ*hns* fragment of C7258 in pWM91	This study
pWM91-*fis*	902 bp *BamH*I-*Spe*I Δ*fis* fragment of C7258 in pWM91	This study
pBBR*lux*	promoterless of *lux*CDABE, Cm^R^	Lab stock
pBBR*lux*-*rfbT*1	554 bp promoter region of *rfbT* in pBBR*lux*	This study
pBBR*lux*-*rfbT*2	403 bp promoter region of *rfbT* without CRP binding site in pBBR*lux*	This study
pBBR*lux-rfbT*1-M1	pBBR*lux*-*rfbT*1 with mutations in the H-NS binding site 1	This study
pBBR*lux-rfbT*1-M3-1	pBBR*lux*-*rfbT*1 with mutations in the middle of the H-NS binding site 3	This study
pBBR*lux-rfbT*1-M3-2	pBBR*lux*-*rfbT*1 with mutations in the front part of the H-NS binding site 3	This study
pGRG25	Transposition plasmid, *ori*T, pSC101 *ori ts*, Amp^R^	McKenzie and Craig (2006)
pGRG25-*hns*	*hns* promotor region and ORF of C7258 clone in pGRG25	This study
pTXB1	Expression vector for construction of in-frame	New England BioLabs
	Fusions with chitin binding domain, Amp^R^	
pTXB1-*hns*	*hns* ORF in expression vector pXTB1, Amp^R^	Wang H. et al. (2012)

### 2.2. Construction of mutants and complementation plasmids

Mutants Δ*hns*, Δ*fis* were constructed by homologous recombination mediated by suicide plasmid using C7258 as a precursor, while Δ*crp*Δ*hns* and Δ*crp*Δ*fis* used Δ*crp* (WL7258) as a precursor. Upstream and downstream chromosomal DNA fragments flanking the *hns* or *fis* were amplified from C7258 genomic DNA using the corresponding primers listed in Table 2. The amplicons were stitched together by overlapping PCR. Δ*hns* or Δ*fis* fragments were cloned into pWM91 and constructed in DH5αλ*pir*. The resulting pWM91-Δ*hns* or pWM91-Δ*fis* was introduced into C7258 or Δ*crp* (WL7258) by conjugation from S17-1λ*pir*. Exconjugants and mutants were selected as described previously (Wu et al., 2015; Li et al., 2019).

**Table 2:** Primers used in this study.

Primer pairs	Oligonucleotide sequences (5′-3′)*	Purposes
*hns*-F1-up- *BamH*I	GCGGGATCCTTCCACAATTCATTGGCATCAC	Δ*hns* deletion strain construction
*hns*-F1-dn	ATCCAAATTGTGAACAGGAATTTTGCCAGA	
*hns*-F2-up	TGAACAGGAATTTTGCCAGAAACTAAAATG	
*hns*-F2-dn-*Spe*I	GGACTAGTACACCGAAGATTCCGCTAAAC	
*fis*-F1-up- *BamH*I	GCGGGATCCGGTGAGGCGGAATACGACAG	Δ*fis* deletion strain construction
*fis*-F1-dn	ACGTCGGTGAAGAATTCGGTCTAGCTCTTC	
*fis*-F2-up	GAAGAGCTAGACCGAATTCTTCACCGACGT	
*fis*-F2-dn-*Spe*I	GGACTAGTAAAGTGGGCGAGTAGGGTTTC	
*hns*-Tn7-*Not*I-up	GCGCGGCCGCTCAAGCGACATCATGTCAAC	C7258Δ*hns::hns* complementation strain construction and identification
*hns-*Tn7*-Xho*I-dn	GCTCTAGATCAGTATCCGTTCGAGTTAA	
*glms*-F	CGATTGCGGTAGAAGCGTC	
*glms-hnsR*	AGACTAAATGAGCCAAATGA	
*thyA*-qPCR-up	ACATGGGACGCGTGTATGG	qPCR for *thyA*
*thyA*-qPCR-dn	ATATGACCACCATCAGGCTTAGC	
*rfbT*-qPCR-up	TTCTTGAAAGCGAATTTGGATTGC	qPCR for *rfbT*
*rfbT*-qPCR-dn	GTGTATATGACGAGCAGCGATTC	
*rfbT*1*-*up*-Sac*I	CCCGAGCTCCGCAACAGAGCAAG ATGT	Construction of *rfbT-lux* reporter plasmids
*rfbT*2*-*up*-Sac*I	CCCGAGCTCTTAGAGCGGACGATCGAG	
*rfbT-*dn*-BamH*I	CGGGATCCGACTGAATAGCATCAAGC	
*rfbT*-*hns*-shift-up	CAAGGATCAGGCAGATATG (5’biotin label)	Probe-*hns*
*rfbT*-*hns*-shift-dn	CTTGCAGATGCAGGTTTGAG (5’biotin label)	
*rfbT-crp-*shift-up	CGTTACTTGAAGCGACTTGT(5′ biotin-labeled)	Probe-N7
*rfbT-crp-*shift-dn	CAAACATATCTGCCTGATCC (5′ biotin-labeled)	
*rfbT*-up (FAM)	CAAGGATCAGGCAGATATG	DNase I footprinting assay
*rfbT*-dn	CTTGCAGATGCAGGTTTGAG	
*rfbT*1-M1-R	GGGTTCGCTCTGTGTGAGGTTCAAACA	Construction of mutant *rfbT-lux* reporter plasmids
*rfbT*1-M1-F	TGTTTGAACCTCACACAGAGCGAACCC	
*rfbT*1-M3-1-R	AATGGATTTGCCATGTGTGTGACATTTAGAAG	
*rfbT*1-M3-1-F	CTTCTAAATGTCACACACATGGCAAATCCATT	
*rfbT*1-M3-2-R	GATTTGCCATTTTAGTTCCATTTAGAAG	
*rfbT*1-M3-2-F	CTTCTAAATGGAACTAAAATGGCAAATC	

*The underlined bases indicate the restriction enzyme sites.

*Chromosomal* complementation strain Δ*hns*::*hns* was constructed using a temperature-sensitive transposable plasmid PGRG25. pGRG25 contains a Tn7 transposon that can carry the target fragment for specific recombination with the chromosome of the host bacterium, directionally inserted into the downstream of the *glms* in the chromosome (McKenzie and Craig, 2006). For this purpose, *hns* promotor region and open reading frame (ORF) was amplified and cloned into pGRG25 to generate pGRG25-*hns* which was mobilized into Δ*hns* by conjugation. The chromosomal insertion of the transposon was induced by 0.1% arabinose in LB broth at 30°C for 16 h, then screened on LB agar by a temperature at 42°C. Grown colonies were tested for Amp sensitivity, and proper insertion of *hns* downstream of *glms* was verified by PCR with primers targeting the *glms* and *hns* sequences. Primer sequences used here are shown in Table 2.

### 2.3. RNA extraction and quantitative reverse transcription PCR

*Vibrio cholerae* strains were cultured to OD_600_ 1.0. Total RNA extraction, removal of chromosomal DNA contamination and cDNA synthesis were performed as previously described (Wu et al., 2015). Equation *R* = 2^− (ΔCq *rfbT*-ΔCq *thyA*)^ was used to calculate the relative expression values (R) of *rfbT*, where Cq is the threshold cycle fraction and *thyA* was used as an internal reference. A control reaction with total RNA as a template was performed for each sample to exclude contamination from chromosomal DNA. Primers used were listed in Table 2.

### 2.4. Transcriptional reporter fusion construction and bioluminescence assay

Two different length fragments of *rfbT* promoter region were, respectively, amplified and cloned into pBBR*lux* upstream of the promoterless *luxCDABE* operon. The resultant fusion plasmids pBBRlux-*rfbT*1 and pBBRlux-*rfbT*2 were constructed in DH5α*λpir* and then mobilized into *V. cholerae* strains C7258 and ∆*hns* by conjugation from S17-1*λpir*. pBBR*lux*-*rfbT*1-M1, pBBR*lux*-*rfbT*1-M3-1, and pBBR*lux*-*rfbT*1-M3-2 reporter fusions containing the specific mutations in the predicted H-NS binding sites were generated by PCR-based site-directed mutagenesis with pBBRlux-*rfbT*1 as a template. Overnight cultures of *V. cholerae* strains containing *lux* reporter fusion plasmid were diluted at 1:100 in fresh LB and incubated at 37°C with shaking to grow to exponential phase. 200 μl of the broth was transferred into 96 well microtiter plates (Costar 3,917) every 1 h, and luminescence and OD_600_ were measured using a microplate reader (Infinite M200 Pro, Tecan, Austria). Luminescence activity was calculated as light unit/OD_600_ as previously described (Pan et al., 2018).

### 2.5. Expression and purification of H-NS protein

**Figure 1:**
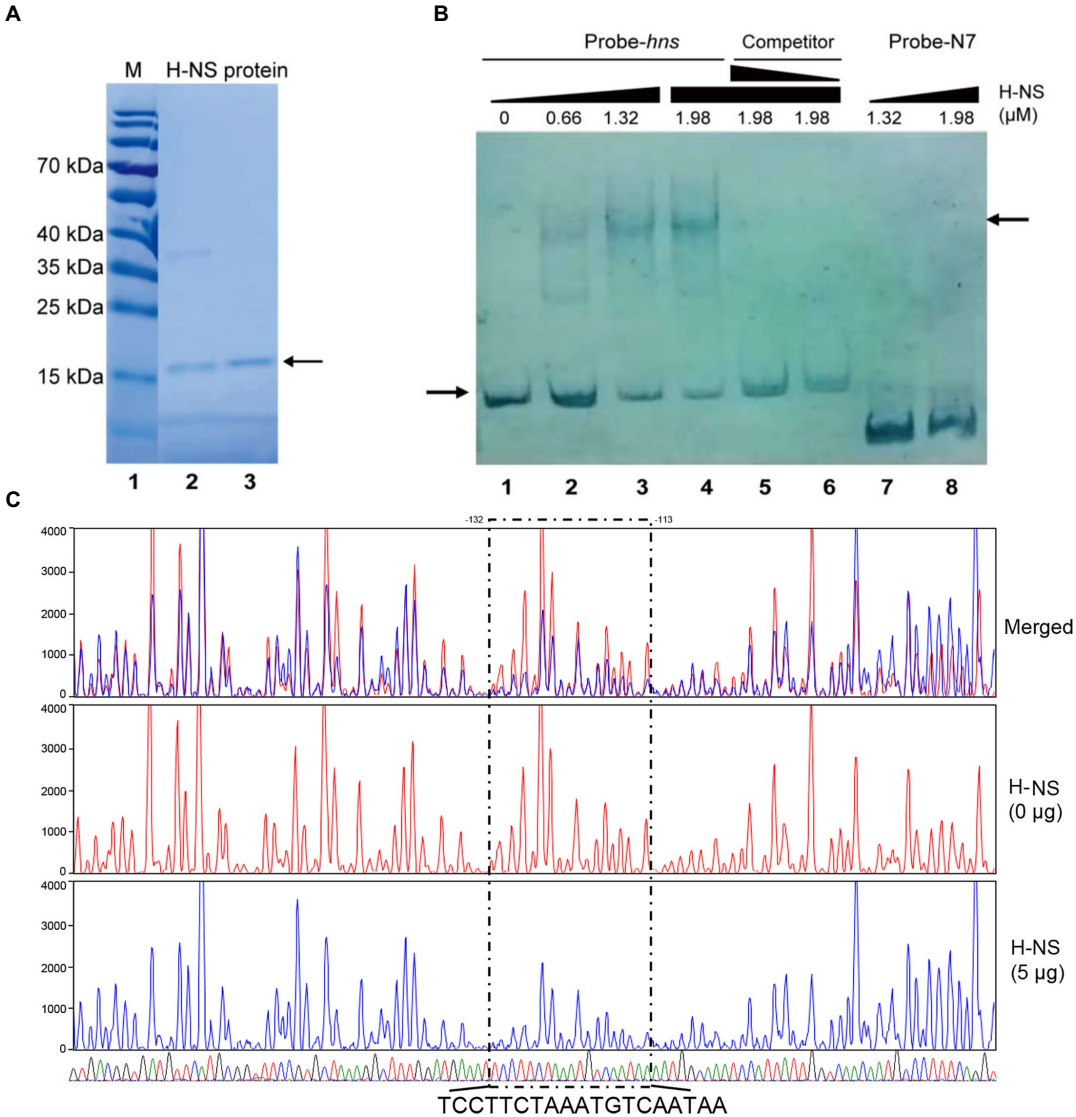
H-NS physically binds to the promoter region of *rfbT*. **(A)** H-NS protein purification. Lane 1 is protein marker, and lanes 2–3 are purified H-NS proteins. The right arrow indicates purified bands. **(B)** EMSA of H-NS bound to *rfbT* promoter regions. EMSA was described in the “Materials and Methods.” Biotin-labeled 256-bp DNA probe (20 ng) was incubated with increasing amounts of purified H-NS protein. For competitive analysis, the identical but unlabelled probe was added at 10 or 50-fold concentration relative to the labeled one (5–6 channels). Lanes 7 and 8 are Probe-N7 with H-NS. The left arrow indicated the free probe, whereas the right arrow referred to H-NS-*rfbT* bound one. **(C)** DNase I footprinting assay of H-NS binding to the promoter region of *rfbT*. As described in the “Materials and Methods,” purified H-NS protein were incubated with FAM-labelled fragments of the *rfbT* promoter region, and then the fragments were digested with optimized DNase I. Finally, the digested fragments were analyzed, and the protected regions were boxed and marked. The colored traces representing the different concentrations of H-NS used (red, 0 μg and blue, 5 μg) are indicated separately and then merged, together with the DNA sequencing results (G, T, A, and C) displayed by four different colors. The region where the blue traces drop is the binding region of H-NS to the probe.

*E. coli* strain ER2566 containing the recombinant expression plasmid pXTB1-HNS (Wang H. et al., 2012) was cultured to OD_600_ of 0.5 with shaking at 37°C and then protein expression was induced with 0.4 mM IPTG for 4 h at 28°C. The cells were collected by centrifugation, resuspended in ice-cold Column buffer (20 mM Tris–HCl, pH 8.0, 0.5 M NaCl, and 1 mM EDTA), and lysed by sonication. The cell debris was removed by centrifugation, and H-NS-intein fusion protein with chitin binding domain (CBD) was purified using IMPACT™ Kit (New England Biolabs, United Kingdom) according to the manufacturer’s instructions. The clarified lysate was slowly loaded onto the equilibrated chitin column, and then the chitin column was washed with 20 bed volumes of Column Buffer. Subsequently, the column was quickly washed with 5 bed volumes of the Cleavage Buffer (Column Buffer containing 80 mM DTT), and then incubated at 4°C overnight for full cleavage reaction on-column. Finally, the H-NS was eluted with Column Buffer. H-NS-containing fractions were combined and dialyzed against Column Buffer at 4°C to remove DTT. The purity of the recombinant H-NS was analyzed by SDS-PAGE (Figure 1A), and the protein concentration was determined by a Pierce BCA protein assay kit (Thermo Fisher Scientific, United States). The protein was stored in 20% glycerol at - 80°C.

### 2.6. Electrophoretic mobility shift assays

Probe-*hns* was a 256 bp fragment of *rfbT* promoter region containing all of the predicted H-NS binding sequences, which was amplified with 5′ biotin-labeled primers. Competing cold probe was amplified using the same primers without a biotin label. Probe-N7 (Li et al., 2019) was a 140 bp fragment of *rfbT* promoter region containing the previously determined CRP binding site and used as a nonspecific control probe for H-NS binding (Li et al., 2019). The reaction mixture of 15 ng biotin-labeled probe with increasing amounts of purified H-NS protein in reaction buffer (10 mM Hepes, 150 mM KCl, 1 mM EDTA, 1 mM DTT, 10 mM (NH_4_)_2_SO_4_, 0.2% Tween 20) together with 100 ng BSA and 100 ng *CF*-DNA in each reaction (20 μl) was incubated at 28°C for 30 min and then separated on a 6% native polyacrylamide gel. The free and H-NS-bound probes were visualized with the Chemiluminescent Nucleic Acid Detection Module (Thermo Fisher Scientific, United States) according to the manufacturer’s instruction after transferring them onto nylon membranes.

### 2.7. DNase I footprinting assay

For preparation of fluorescent FAM-labeled probes, the promoter region was PCR amplified with 2 × HIFI DNA polymerase premix from the plasmid pBBR*lux*-*rfbT*1 using primers of *rfbT*-up (FAM)and *rfbT*-dn. The FAM-labeled probes were purified by the Wizard® SV Gel and PCR Clean-Up System (Promega, United States) and were quantified with NanoDrop 2000C (Thermo, United States).

DNase I footprinting assay was performed as previously described (Wang Y. et al., 2012). For each assay, 250 ng probes were incubated with different amounts of protein in a total volume of 40 μl. After incubation for 30 min at 25°C, 10 μl solution containing about 0.015 unit DNase I (Promega, United States) and 100 nmol freshly prepared CaCl_2_ was added and further incubation was performed at 37°C for 1 min. The reaction was stopped by adding 140 μl DNase I stop solution (200 mM unbuffered sodium acetate, 30 mM EDTA, and 0.15% SDS). Samples were first extracted with phenol/chloroform, and then precipitated with ethanol. Pellets were dissolved in 30 μl MiniQ water. The preparation of the DNA ladder, electrophoresis, and data analysis were the same as described before (Wang Y. et al., 2012), except that the GeneScan-LIZ600 size standard (Applied Biosystems) was used.

### 2.8. Statistical analysis

GraphPad Prism 9 software was used for statistical analysis and graphical representation of data. Statistical significance was determined by an unpaired two-tailed Student’s *t*-test.

## 3. Results

### 3.1. Characterization of the promoter region of *rfbT*

**Figure 2:**
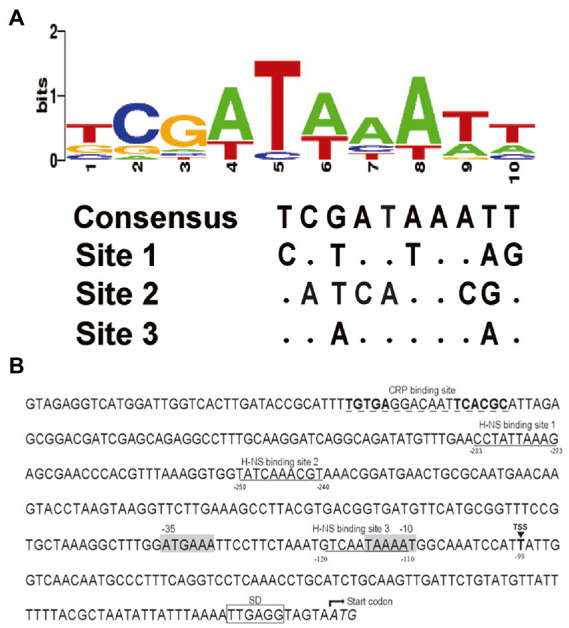
Nucleotide sequence analysis of the *rfbT* promoter region. **(A)** Conservation analysis of H-NS binding sites. Virtual Footprint was used to predict the binding sites of H-NS at the *rfbT* promoter (https://www.prodoric.de/vfp/). **(B)** Characteristics of the *rfbT* promoter region. Underlined sequences show the H-NS binding site predicted by Virtual Footprint. Dashed underlined sequences show the CRP binding sites characterized previously (Li et al., 2019). The black box marks the Shine-Dalgarno sequence (SD) (Ito et al., 1991). The triangle and the bold T represent the proved TSS of the *rfbT*, and the gray shaded sequences represent its −10 and −35 regions (Stroeher et al., 1992). The negative numbers at the bottom of sequences indicate the nucleotide positions relative to the start codon of *rfbT*.

Though the gene *rfbT* has been recognized as the genetic determinant of Ogawa serotype of *V. cholerae* O1 serogroup for more than 30 years (Stroeher et al., 1992) and various kinds of mutations were revealed in the *rfbT* coding sequence of isolates from different space–time sources (Sharifnia et al., 2012; Liang et al., 2013; Karlsson et al., 2016), its regulation and the molecular structural features except the transcriptional start site, putative −35 and − 10 elements of the promoter-regulatory region remain unclear. Previously, we identified a *cis*-regulatory element, i.e., a non-canonical CBS in the promoter region, through which global regulator CRP exerts an activational effect (Li et al., 2019). Further sequence analysis revealed that the G + C content of promoter-intergenic region *of rfbT* (41.2%) is quite low compared with the *V. cholerae* genome in general (47.7% for chromosome I and 46.9% for chromosome II). In other words, the promoter-intergenic region of *rfbT* is AT rich and probably prone to be regulated by small nucleoid associated proteins such as H-NS and Fis which tend to bind AT rich sequences. Indeed, subsequent Virtual Footprint and PRODORIC analysis using the 10-bp H-NS consensus (Bouffartigues et al., 2007; Figure 2A) revealed 3 potential H-NS binding elements, site 1 (5’-CCTATTAAAG-3′), site 2 (5’-TATCAAACGT-3′), and site 3 (5’-TCAATAAAAT-3′) in the *rfbT* promoter-intergenic region (Figure 2B). The three binding sites are, respectively, located at nucleotides −283 to −273, −250 to −240, and − 120 to −110 relative to the *rfbT* start codon, and are all downstream of nonclassical CBS (Figure 2B). Of these, 8 of the 10 bp at site 3 is consistent with the consensus, followed by site 1 with 5 bp, and finally site 2 with only 4 bp (Figure 2A). It’s worth noting that the site 3 overlaps the predicted −10 promoter element (Figure 2B). These findings strongly indicated the possibility that H-NS regulates serotype-shifting gene *rfbT* expression.

### 3.2. H-NS negatively regulates *rfbT* expression

**Figure 3:**
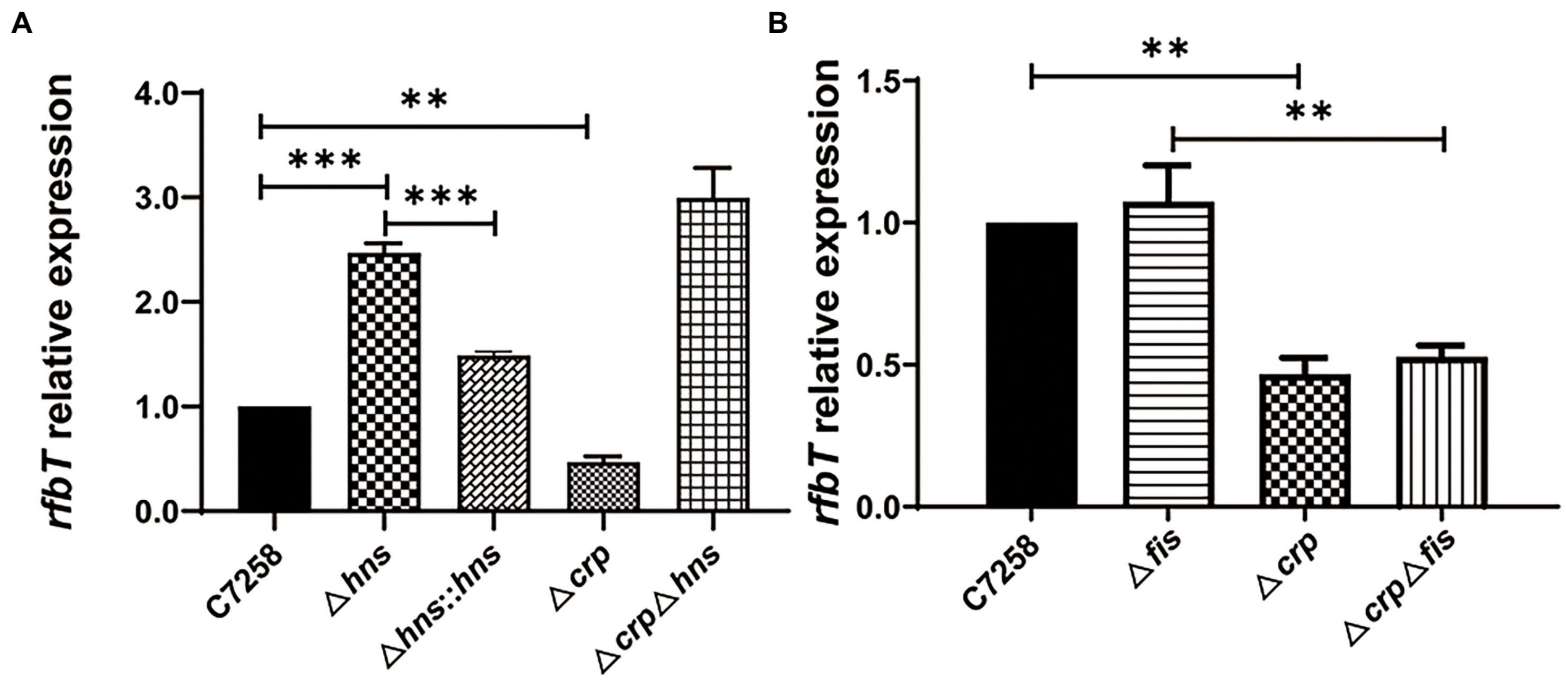
Effects of H-NS, CRP and Fis on *rfbT* expression. The mRNA abundances around OD_600_ of 1.0 were determined using qPCR. **(A)** Comparison of mRNA level of *rfbT* in *V. cholerae* strains C7258, Δ*hns*, Δ*hns::hns*, Δ*crp*, and Δ*crp*Δ*hns*. The equation *R* = 2^−(ΔCq *rfbT* - ΔCq *thyA*)^ is used to calculate the mRNA levels. **p < 0.01, ***p < 0.001. **(B)** The relative mRNA levels of *rfbT* in C7258, Δ*fis,* Δ*crp*, and Δ*crp*Δ*fis*. The calculation method is the same as **(A)**. **p < 0.01.

To determine whether H-NS is involved in the regulation of *rfbT*, we constructed an *hns* deletion mutant using C7258 as a precursor and detected the *rfbT* mRNA level in WT C7258, and Δ*hns* mutant. As shown in Figure 3A, compared to the C7258, Δ*hns* mutant statistically produced more *rfbT* mRNA. To further confirm the result, we constructed an *hns* complementation strain C7258Δ*hns*::*hns* where a single copy of *hns* gene with its native promoter region was integrated downstream of chromosomal *glms*. As displayed, the *rfbT* mRNA abundance was reduced in C7258Δ*hns*::*hns* compared to Δ*hns* mutant, though the expression level was not restored to the WT level (Figure 3A). Together, these results showed that H-NS negatively regulates *rfbT* expression.

In our previous study, we demonstrated that CRP could positively regulate *rfbT* transcription, and then we wondered whether H-NS regulates *rfbT* expression in a CRP-dependent manner (Li et al., 2019). For this purpose, we constructed a Δ*crp*Δ*hns* double mutant and compared its *rfbT* mRNA expression with those of Δ*hns* and Δ*crp* mutants. We found that, contrary to the Δ*crp* mutant where *rfbT* expression was obviously lower than its WT and Δ*hns*, the *rfbT* mRNA level was significantly increased in Δ*crp*Δ*hns* (Figure 3A), and additionally, the Δ*hns* and Δ*crp*Δ*hns* mutants displayed roughly similar *rfbT* mRNA level. These results proved that H-NS negatively regulates the expression of *rfbT* in a CRP-independent manner. From another perspective, these results also indicated that CRP is not required for *rfbT* activation in H-NS negative background.

### 3.3. Fis does not affect *rfbT* expression

Like H-NS, Fis belongs to the small family of nucleoid-associated proteins and is widely implicated in the control of gene expression through binding to the A−/AT-tracts-constituted binding site (Cho et al., 2008). To find out whether Fis takes part in the regulation of *rfbT*, we constructed Δ*fis* and Δ*crp*Δ*fis* deletion mutants and measured the *rfbT* mRNA levels. As displayed in Figure 3B, the *rfbT* mRNA level in Δ*fis* is similar to the WT, and deletion of *crp* significantly reduced *rfbT* expression regardless of the presence or absence of *fis*, implying that Fis is not involved in the regulation of *rfbT*.

### 3.4. H-NS represses the promoter activities of *rfbT*

**Figure 4:**
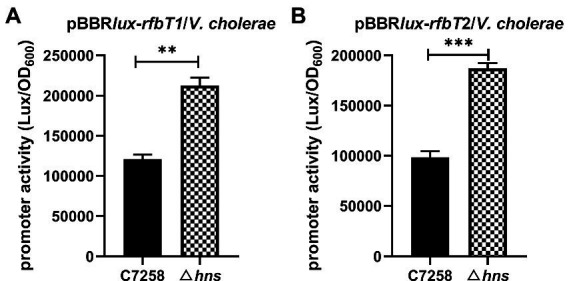
Luminescence activities of *lux* reporter fusion plasmids in *V. cholerae* C7258 and Δ*hns*. **(A)** Luminescence activities of pBBRlux-*rfbT*1 in C7258 and Δ*hns*. *rfbT*1 is a 554 bp fragment of *rfbT* promoter region. **(B)** Luminescence activity of pBBRlux-*rfbT*2 in C7258 and Δ*hns*. *rfbT*2 is a 403 bp fragment of the *rfbT* promoter region without CRP binding site. The luminescent activities were reported as luminescence/OD_600_ at the designated time points. ***p < 0.001.

To determine whether the H-NS-mediated repression of *rfbT* occurs at transcription level, we constructed two transcriptional reporter plasmids by fusing the different length fragments of promoter region of *rfbT* to the promoterless bioluminescence reporter genes *luxCDABE*. The 552-bp promoter fragment in pBBR*lux*-*rfbT*1 contains both the previously identified CBS and the predicted H-NS binding sites, while the 403-bp promoter region in pBBR*lux*-*rfbT*2 lacks the CBS. Consistent with the *rfbT* mRNA expression, bioluminescence activities of both pBBR*lux*-*rfbT*1 and pBBR*lux*-*rfbT*2 in Δ*hns* were significantly higher than in its WT (Figure 4). These results demonstrated that H-NS negatively regulates *rfbT* at the promoter level. H-NS represses the transcription of *rfbT* probably through binding to the predicted binding sites and therefore the direct interaction still needs to be clarified.

### 3.5. H-NS directly binds to the promoter of *rfbT*

To verify the direct binding of H-NS to the *rfbT* promoter region, we performed EMSA with purified H-NS protein. A 256-bp DNA fragment of *rfbT* promoter region encompassing the three predicted H-NS binding sites were labeled with biotin at 5′ end and used as specific probe. The same fragment without biotin label was employed as competing cold probe. The 135 amino acids *V. cholerae* H-NS protein was induced and purified from pTXB1-HNS recombinant plasmid (Wang H. et al., 2012) in *E. coli* host ER2566 and reached a purity of higher than 90% (Figure 1A). As displayed in Figure 1B, inclusion of H-NS (0.66 μM) generated two shifted bands with slower mobility. With the increment of H-NS (>1.32 μM), the intensity of the higher-shifted band was increased, concomitantly with the decrease of the amount of free probe. Adding the same, but unlabeled 256-bp DNA fragment greatly competed with the labeled probe in a dose-dependent manner. Both of the 10-fold and 50-fold addition of the competing cold probe completely abolished the shifted-band, and concurrently the labeled probe was released as the free one. As expected, the amount of the released free probe is more in 50-fold cold probe reaction mixture than the 10-fold one. To further validate the specificity of H-NS binding, we adopted Probe-N7 which is a 140-bp DNA fragment of *rfbT* promoter region containing the intact CBS but without the predicted H-NS binding element (Li et al., 2019). No apparent shifted band was observed even when Probe-N7 was incubated with higher amount of H-NS (1.32 μM or 1.98 μM) under the same reaction condition, indicating that H-NS could not bind to the *rfbT* promoter fragment lack of specific binding site.

To further clarify the real H-NS binding site in the *rfbT* promoter and dissect the binding sequences, we conducted DNase I footprinting analysis. As shown in Figure 1C, the assay revealed one clearly protected region against DNase I digestion which is composed of TCCTTCTAAATGTCAATAA extending from −132 to −113 relative to the start codon of *rfbT*, i.e., this region completely overlaps the predicted binding site 3 extending from −120 to −110. Though EMSA displayed two H-NS retarded bands that seems somehow in accordance with the predicted existence of two more conserved binding sites 1 and 3, unexpectedly, the less conserved binding site 1 was not confirmed in the DNase I footprinting assay, which suggested that the more conserved site 3 is intrinsically the real binding site with high-affinity. Of course, we cannot exclude the possibility that the current assay condition did not favor binding to the low-conservation site 1.

**Figure 5:**
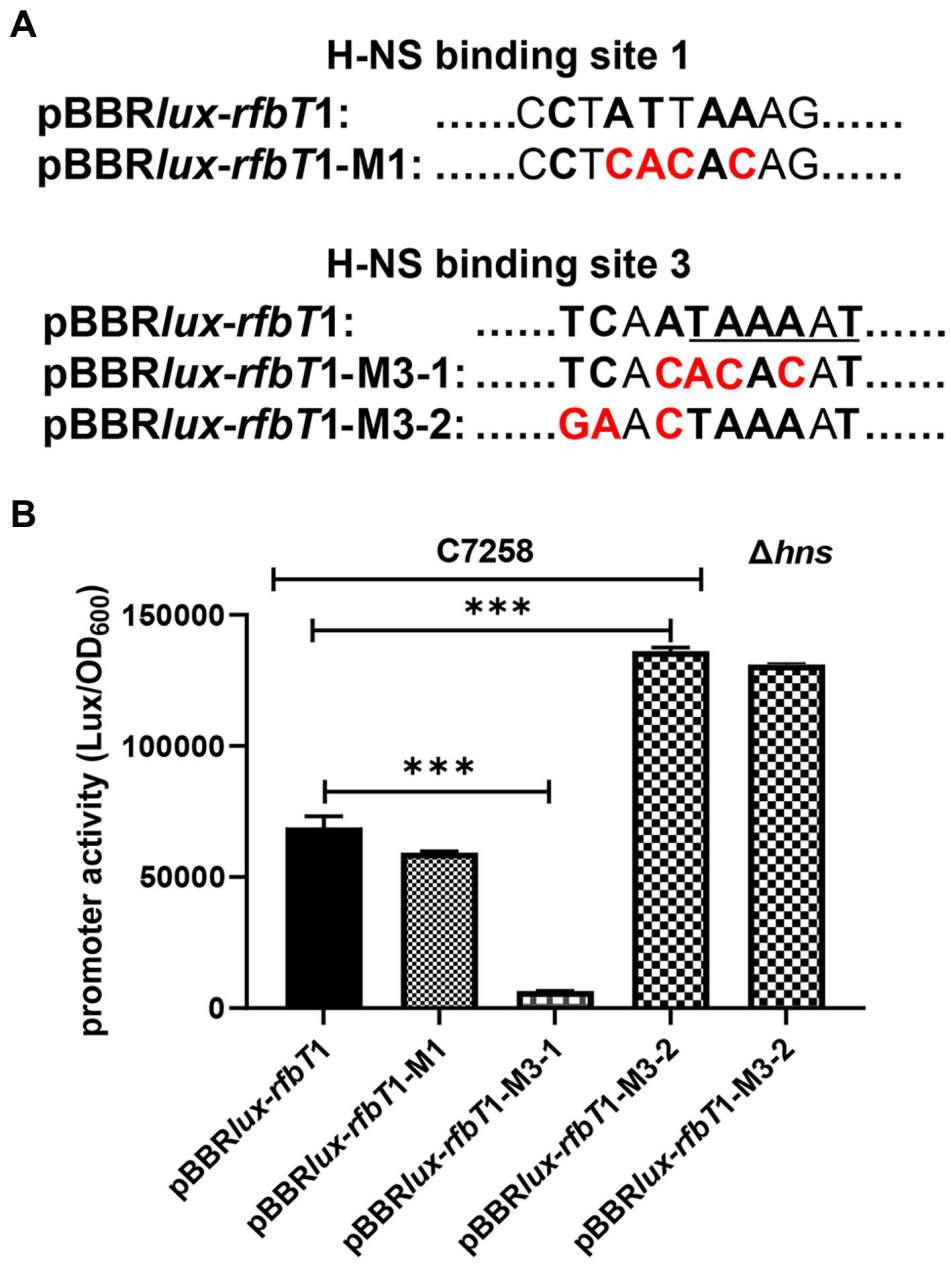
Schematics of the site-directed mutagenesis of the H-NS binding sites at *rfbT* and luminescence activities of *lux* reporter fusion plasmids containing mutations in *V. cholerae*. **(A)** The pBBR*lux*-*rfbT*1-M1, pBBR*lux*-*rfbT*1-M3-1, and pBBR*lux*-*rfbT*1-M3-2 were constructed by introducing specific mutations in the relevant H-NS sites at the *rfbT* promoter. The bases identical to the 10-bp H-NS binding consensus were indicated in boldface letters and the changed ones in red color. The underlined sequence is the predicted −10 motif of *rfbT* promoter. **(B)** Luminescence activities of pBBR*lux*-*rfbT*1, pBBR*lux*-*rfbT*1-M1, pBBR*lux*-*rfbT*1-M3-1, and pBBR*lux*-*rfbT*1-M3-2 in C7258 and pBBR*lux*-*rfbT*1-M3-2 in Δ*hns*. The luminescence activities were reported as luminescence/OD_600_ as described in the “Materials and Methods.” ***p < 0.001.

To further validate that site 3 is the real functional H-NS binding site and the less conserved site 1 has no function, we set out to introduce mutations at sites 1 and 3 through PCR-based site-directed mutagenesis with pBBRlux-*rfbT*1 as the template (Figure 5A). We introduced 4-bp changes in the site 1 by replacing the ATT and the antepenultimate A with CAC and C, respectively, to generate a new construct pBBRlux-*rfbT*1-M1. Considering that site 3 overlaps the predicted −10 promoter element, we introduced two sets of mutations to construct pBBR*lux*-*rfbT*1-M3-1 and pBBR*lux*-*rfbT*1-M3-2. In pBBR*lux*-*rfbT*1-M3-1, the conserved ATA and the antepenultimate A were changed to CAC and C, where TA and A are involved in the −10 motif of *rfbT* promoter. In pBBR*lux*-*rfbT*1-M3-2, the TC and second A were changed to GA and C. These new constructs were mobilized into *V. cholerae* WT and Δ*hns* to measure the corresponding bioluminescence activities. As depicted in Figure 5B, pBBRlux-*rfbT*1-M1 and pBBRlux-*rfbT*1 had similar bioluminescence activities in the WT background which indicated that the mutation of sites 1 has no effect on the *rfbT* promoter, i.e., site 1 is indeed not a H-NS binding site. However, compared to the pBBRlux-*rfbT*1, pBBR*lux*-*rfbT*1-M3-2 displayed significantly high bioluminescence activity in WT as same as in the Δ*hns*, implying that the introduced mutation affects H-NS binding and thus relieves its repression. Not surprisingly, pBBR*lux*-*rfbT*1-M3-1 almost completely lost the bioluminescence signal due to the mutations that destroyed the −10 motif of *rfbT* promoter by changing TAAAAT to ACACAT. Altogether, these results further experimentally validated that site 3 partially overlaps the −10 motif of *rfbT* promoter and is the intrinsically functional H-NS binding site.

## 4. Discussion

*rfbT* (also named *webT* or *tsfB*) is a genetic determinant of Ogawa serotype of *V. cholerae* O1 by encoding an enzyme that methylates the O-PSs-terminal peraminoglycans of surface LPS. Genetic alteration of *rfbT* results in impaired function of the enzyme, causing serotype shift from Ogawa to Inaba. Various mutational events of *rfbT* leading to serotype switching have been reported under different circumstance around the world (Longini et al., 2002; Liang et al., 2013; Karlsson et al., 2016). The epidemiological significance of the two serotype variants shift, the influencing factors and regulation mechanism remain largely uninvestigated. Driven by the serotype-specific immunity acquired within the infected host population (Longini et al., 2002) is a commonly accepted speculation.

In our previous study, we reported the global regulator CRP activates the expression of *rfbT* and further revealed the underlying genetic mechanism (Li et al., 2019). In this study, we reported another global regulator H-NS which is involved in the transcriptional repression of *rfbT* by directly binding to its AT-rich promoter region (Figures 1, 3–5). Though more than one potential binding sites were predicted, binding site 3 was finally validated to be the functional H-NS binding site by DNase I footprinting assay and site-directed mutagenesis (Figures 1C, 5). This site overlaps the −10 promoter element (Figures 1C, 2B, 5), thus strongly implying that H-NS functions to block *rfbT* transcription by interfering with RNA polymerase binding and activity. In addition, we showed that though CRP activates the transcription of *rfbT* (Li et al., 2019), it loses this function in the H-NS minus background (Figures 3A, 4), indicating that CRP may act as an antisilencer by antagonizing H-NS repression in the *rfbT* promoter. A number of virulence regulators, such as ToxT, ToxR, and IHF have been reported to act as antirepressors by displacing H-NS at specific promoters (Stonehouse et al., 2008, 2011; Kazi et al., 2016). In these cases, the H-NS binding sites generally overlap or are adjacent to the binding sites of specific activators, such as at *V. cholerae tcpA* promoter, the binding site of IHF is located between the two sites of H-NS with 6 bp intervals with the up one and overlapping the down one (Stonehouse et al., 2008). At cholera toxin *ctx* promoter, competitive binding to the overlapping H-NS/ToxT binding sites was proved in EMSA where ToxT could displace H-NS from the *ctx* promoter (Stonehouse et al., 2008). However, in our case, the detailed molecular mechanism of CRP antagonizing H-NS repression on *rfbT* remains investigated considering the CRP-specific CBS and H-NS binding site 3 are separated by 220 bp. We reasoned the alleviation of H-NS repression by CRP at *rfbT* is mechanistically distinct from *tcpA* and *ctx* and will be investigated in the future.

Selective silencing of horizontally acquired genes is a common theme in H-NS transcription regulation (Navarre et al., 2006). Horizontally acquired foreign DNA generally has a lower GC-content than its progenitor genome (Kazi et al., 2016). The G + C content of *rfbT* (31.7%) is quite low compared with the rest of the *rfb* region (39.1%) and with *V. cholerae* genome (47% in average). These observations suggest that *rfbT* is acquired as a foreign DNA and its expression is normally silenced by H-NS. This repression effect could be alleviated by other regulators such as CRP under appropriate environmental conditions. The preference for binding low GC-content DNA is also shared by IHF and Fis. Fis has been proved to be unable to affect the expression of *rfbT*, while the regulation effect of IHF remains to be determined.

The silencing function of H-NS is dependent on its oligomerization properties. It is believed that an H-NS dimer is the minimal functional binding unit (Badaut et al., 2002). Environmental stimuli such as temperature and osmolality can alter the oligomerization states of H-NS *in vivo* and hence affect its gene-silencing properties (Amit et al., 2003; Stella et al., 2006; Bouffartigues et al., 2007). At a certain osmolality, the ability of H-NS to bind DNA decreased significantly with increasing temperature (Amit et al., 2003; Bouffartigues et al., 2007). In a word, a variety of factors can affect the function of the H-NS. Whether these environmental factors affect the phenotype of Ogawa serotype strain through H-NS remains to be clarified.

In summary, we demonstrated that *V. cholerae* Ogawa serotype specific gene *rfbT* is transcriptionally repressed by the global regulator H-NS through directly binding to a specific *cis* regulatory element in the promoter region. This work expanded our knowledge of understanding the genetic determinants and complicated regulatory mechanism of *V. cholerae* O1 serotype shift.

## Data availability statement

The original contributions presented in the study are included in the article/supplementary material, further inquiries can be directed to the corresponding authors.

## Author contributions

WL and BK conceived and designed this study. YH, JL, HG, XL, and RD contributed to the experiment. YH and WL contributed to writing the manuscript. All authors contributed to the article and approved the submitted version.

## Funding

This study is supported by the National Key R&D Program of China under grant 2021YFC2300302.

## Conflict of interest

The authors declare that the research was conducted in the absence of any commercial or financial relationships that could be construed as a potential conflict of interest.

## Publisher’s note

All claims expressed in this article are solely those of the authors and do not necessarily represent those of their affiliated organizations, or those of the publisher, the editors and the reviewers. Any product that may be evaluated in this article, or claim that may be made by its manufacturer, is not guaranteed or endorsed by the publisher.
